# Gravid Uterus in an Umbilical Hernia

**DOI:** 10.1155/2012/439489

**Published:** 2012-07-02

**Authors:** Lawrence C. E. Mbuagbaw, Frederick L. I. Morfaw

**Affiliations:** ^1^Centre for the Development of Best Practices in Health, Yaoundé Central Hospital, P.O. Box 87, Henri Dunant Avenue, Messa, Yaoundé, Cameroon; ^2^Department of Obstetrics and Gynaecology, Faculty of Medicine and Biomedical Sciences, University of Yaoundé 1, P.O. Box 1364, Yaoundé, Cameroon

## Abstract

Umbilical hernias large enough to contain a gravid uterus are rare. We report a case of a woman with prolapse of a gravid uterus through a previously repaired umbilical hernia. Our plans for elective surgery with caesarean section and hernia repair were foiled by poor compliance. The hernia was repaired during an emergency caesarean section. We provide details of her management and briefly review the literature on umbilical hernias and pregnancy. Surgical management offers an opportunity for hernia repair and can ensure a safe delivery for the mother and child.

## 1. Introduction

Among the less common complications of pregnancy are large umbilical or incisional hernias. Reports of a wide variety of organs protruding through abdominal hernias during pregnancy have been made. Authors have described incarceration of bowel, pedunculated fibroids, and abdominal pregnancies [1–4]. Cases of abdominal wall rupture have even been reported [5]. Most of these cases occur in multiparous women. The following is the case report of a gravid uterus herniating through a large umbilical hernia in a primiparous patient.

## 2. Case Presentation

A twenty-year-old Cameroonian woman, gravida 1 para 0 presented at 35 weeks gestation to the antenatal consultation unit of our hospital with an unusually distended abdomen, for her first antenatal care visit. Her past medical history revealed a herniorrhaphy three years prior to this consultation, for a congenital umbilical hernia.

**Figure 1:**
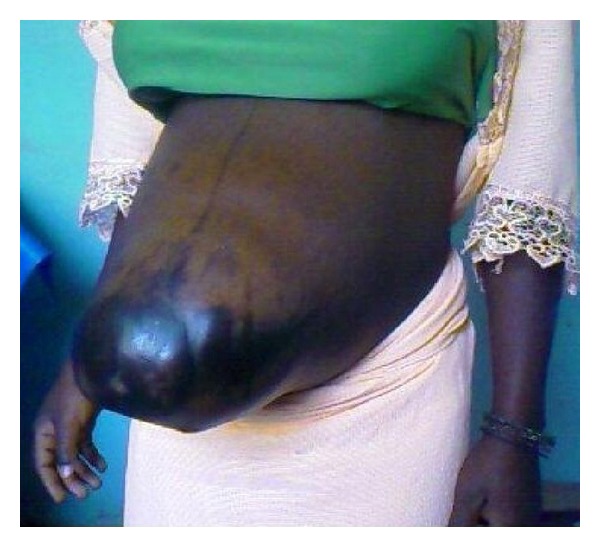
Necrosis and ulceration at tip of hernia.

On physical examination, the patient's general condition was satisfactory. She was afebrile with a pulse rate of 70 beats per minute and a blood pressure of 100/60 mmHg. She had moderately colored conjunctivae and her cardiopulmonary examination was normal. Her abdomen was unduly distended around the central area, with a hyperpigmented necrotic scar overlying the paraumbilical area. Abdominal palpation revealed complete absence of abdominal wall around the paraumbilical area overlying the uterus. This hernia orifice was about 15 cm by 10 cm. Most of the gravid uterus protruded via this orifice in a standing position (Figure 1). The fundal height was estimated at 32 cm. The foetal lie was longitudinal, and foetal parts could easily be palpated across the herniated gravid uterus. Foetal heart sounds were regular and there was no evidence of uterine contractions. The skin around the hernial sac was hyperpigmented, necrotic, and very tense. Vaginal examination revealed a firm, long, median, and closed cervix; the rest of the clinical examination was unremarkable.

Blood and urine analysis were within normal limits. Our hospital did not dispose of an ultrasound machine for further foetal evaluation.

We discussed the possibility of hospitalisation and an elective caesarean section but the patient declined the proposal due to financial constraints and chose to return home against medical advice. Topical antiseptics were given to the patient to apply on the abdominal skin, and the next visit scheduled in one week.

She however presented three weeks later at the maternity in active labour. Clinical evaluation revealed foetal bradycardia with a foetal heart rate at 90 beats per minute. An emergency transverse lower segment caesarean section was performed under general anaesthesia (for acute foetal distress). The incision was paramedian, avoiding the necrotic zones. Just beneath the skin was a large round umbilical hernial sac containing the gravid uterus. We found remnants of the chromic catgut sutures used to repair the umbilical hernia three years ago. We did a transverse lower segment incision on the uterus extracting a live male foetus weighing 3400 g. The APGAR scores were 5 and 7 at the first and fifth minutes, respectively. The placenta was anterior corporeal. Following satisfactory haemostasis on the uterus, we concomitantly repaired the hernial orifice. This was done by excision of all zones of necrotic tissue, reduction of the hernia sac, end-to-end approximation of the orifice borders, and reinforcement of the rectus abdominis muscles. Excess and necrotic skin was removed before closure of the skin. The surgery lasted close to two hours. The postoperatory period was uneventful; the wound healed by primary intention, and the patient was discharged on the eighth day postoperation. She was seen six weeks later during her postpartum visit. She was enjoying relatively good health, her scar was well healed, and her baby was doing well.

## 3. Discussion

The reported prevalence of umbilical hernia is about 15% among pregnant West African women [6]. This fairly common finding may pose a serious obstetric threat necessitating urgent intervention [7]. The possible complications include preterm labour, abortion, strangulation, haemorrhage, intrauterine growth restriction, intrauterine foetal death, dysfunctional labour, rupture of the lower uterine segment, and postpartum haemorrhage amongst others [8]. They are usually associated with previous midline sub-umbilical incisions [9]. In this case, the causative factor was a congenital umbilical hernia.

Therapeutic measures should factor in foetal viability and the associated morbidity and mortality of the mother. Management of incisional hernia in pregnancy is mainly conservative [7]. The therapeutic options include a caesarean section which offers a means of hernia repair during surgery, as opposed to vaginal birth which involves delayed repair [7]. However, given the uncertainties about the integrity of the anterior abdominal wall during labour especially in the case of an incisional hernia, elective caesarean section is considered by many obstetricians as the safest mode of delivery [7]. During these caesarean sections, concomitant herniorrhaphy can be carried out [8]. Nevertheless, elective postpartum herniorrhaphy is reported as the normal standard [10], the reason being that the overstretched abdominal wall may limit proper repair, and the associated risk of wound dehiscence and infection may be higher [11]. Our patient already had an indication for surgery (foetal distress) and was likely to be noncompliant (attending her first antenatal visit at 35 weeks and missing a scheduled appointment).

Pregnancies of this kind must be handled with care. Accrued foetal monitoring, care of necrotic skin and preparation for caesarean delivery are important aspects of management. It may be worthwhile to investigate conservative means of reducing and containing the prolapsing organ within the abdominal cavity until delivery in cases far from term in order to maximize chances of foetal survival. Young et al. [12] report a case of an abbreviated course of steroids for 24 hours prior to caesarean delivery in a patient at 34 weeks gestation with an incisional hernia complicated by bowel obstruction.

Reports of subsequent pregnancies after herniorrhaphy following herniation of a gravid uterus are uncommon in the literature. Given the young age of our patient, we believe she would get pregnant again. Yet in a context where communication is poor and loss to followup is frequent, it may be difficult to obtain further information about this patient.
